# Contribution of Leisure Context, Motivation and Experience to the Frequency of Participation in Structured Leisure Activities among Adolescents

**DOI:** 10.3390/ijerph19020877

**Published:** 2022-01-13

**Authors:** Matea Belošević, Martina Ferić

**Affiliations:** Laboratory for Prevention Research (PrevLab), Department of Behavioural Disorders, Faculty of Education and Rehabilitation Sciences, University of Zagreb, Borongajska Cesta 83f, 10000 Zagreb, Croatia; martina.feric@erf.unizg.hr

**Keywords:** adolescents, context, motivation, experience, frequency, structured leisure time

## Abstract

Leisure time is considered an important context for adolescent development. The purpose of this article is to investigate what contributes to the frequency of adolescents’ participation in structured leisure activities (SLA). Participants were aged 14–21 years (M = 18.87, SD = 1.23) and 44.8% of participants were female. Hierarchical regression analyses were conducted. Results indicate that boys and adolescents who perceive the context of participation in SLA as safe, are externally or intrinsically motivated, and perceive that participation has contributed to their identity development and experiences of initiative, as well as their experiences of stress, are more likely to participate frequently in SLA. On the other hand, these findings indicate that girls and adolescents who are unmotivated to participate in SLA and who experience negative peer influences while participating in SLA are less likely to frequently participate in SLA. It can be concluded that it is important to think much more broadly than just the setting of the activities themselves when promoting young people’s participation in SLA. Some of the features of SLA that promote positive youth development are presented in this paper.

## 1. Introduction

Leisure time is one of the most important aspects of adolescents’ lives and is considered an important context for adolescent development. Leisure activities are things that people do in their leisure time, such as riding a bike or meeting with friends [1,2]. Studies show that adolescents spend a significant part of the day engaged in leisure activities [3]. In Western industrialized countries, adolescents spend nearly half of their “awake” time in leisure [4,5]. In terms of frequency of participation in leisure activities, research shows that young people’s lifestyles have become sedentary [6]. The digital age has brought drastic changes and more and more young people are spending their leisure time on devices. Data from 2015. collected in the UK shows that young people spend 1/3 of their free time (about 14 h per week) on devices and spend less time on sports and cultural activities [7]. Among children and young people, higher levels of sedentary behavior are associated with the following poor health outcomes: increased adiposity; poorer cardiometabolic health, fitness, behavioral conduct/prosocial behaviors and reduced sleep duration [8]. The World Health Organisation [8] recommends that children and adolescents should limit the amount of time they spend sedentary, especially the amount of time they spend in front of screens during their leisure time.

Leisure time can be defined in objective and subjective terms [9,10]. The objective term refers to time spent away from duties (e.g., work, schoolwork, homework) or engaged in certain activities, while the subjective term refers to a ‘state of mind’, i.e., a state of being or a meaningful experience [11]. Although leisure is a time when a person pursues activities that correspond to his or her own interests [12], structured leisure activities (SLA) are characterized by a clear structure with defined rules and goals, are supervised by adults, have a regular schedule, and focus on skill building [13,14,15].

There is debate among experts as to whether all leisure activities contribute to positive adolescent development or whether there are certain characteristics of leisure that must be met for leisure to be considered a context that promotes positive adolescent development. Some authors claim that structured leisure activities, as opposed to unstructured, informal, and passive leisure activities, contribute to positive adolescent development [1,16,17,18,19]. Unstructured leisure activities are thought to have no impact on the development of skills and competencies and therefore do not contribute to positive developmental outcomes for young people [1,16]. In addition, some studies show results that participation in unstructured activities is correlated with substance abuse, sexual risk behaviors, and poor academic performance [16,17,19,20,21,22,23] or is less developmentally beneficial [18,19,20,21,22,23,24,25,26]. Regarding participation in SLA, studies show that it is positively correlated with improved adolescent well-being and progress [27,28,29,30,31,32,33], positively correlated with high levels of self-respect, self-esteem, life satisfaction, and psychological functioning, as well as better physical and mental health in adolescents [18,24,26,28,31,34] and a lower likelihood of adolescent engagement in risk behaviors [17,23,31,35,36,37,38].

However, most authors call for caution in generalizing, given that there are many leisure activities that are not structured and still contribute to the development of youth competencies [14,16,25,39,40] as there are various ways adolescents can spend their leisure time that no one would describe as meaningless or negative (e.g., taking walks in nature, reading a book). For example, productive leisure time has been shown to be a protective factor for youth substance use [10,41,42,43].

Caldwell [42] notes that most of the literature on leisure has focused on the structure of the activity so that distinctions could be made between the importance of organized or structured activities and the negative outcomes of unstructured activities. It seems more important to consider the leisure time context [25,44] and the experiences that adolescents have through participation in leisure activities [1,2,44] rather than viewing leisure activities as structured and unstructured and assessing their contribution to adolescent’s positive development.

A model that takes into account the activity itself, the context, and the leisure experience is the Leisure Activity-Context-Experience Model (LACE model). This model provides a framework that focuses on understanding adolescent development through leisure [2,45]. Caldwell [42] argues that activity, context, and experience are the elements that are interrelated, and their interaction leads to positive or negative leisure experiences [25,46].

Leisure activities, as defined earlier, can be structured and unstructured. In addition, leisure activities occur within a context, meaning that there are elements within an activity or environment that contribute to leisure participation [1,2]. Eight contextual elements of structural activities have been identified that must be present to promote positive developmental outcomes: physical and psychological safety, appropriate structure, supportive relationships, opportunities for belonging, positive social norms, support for efficacy and meaning, opportunities for skill building, and family, school, and community integration [25,42,47].

Furthermore, to understand how leisure contributes to adolescent development, consideration should also be given to the experiences adolescents have through participation in leisure activities [1,2]. These experiences gained through participation in leisure activities may be positively or negatively correlated with an individual’s personal dimensions, depending on the type of leisure activity in which an individual participates and/or situational features of the leisure context [44]. For example, engagement, interest, and motivation are associated with positive outcomes such as initiative, self-efficacy, and skill acquisition, thus contributing to positive adolescent development. In contrast, negative experiences (boredom, stress, and conflict) are associated with negative outcomes such as substance abuse and delinquency [10,13,25,41,44,47]. simply participating in leisure activities without interest or motivation does not necessarily lead to positive developmental outcomes for adolescents. Interest, intrinsic motivation, and flow are three leisure experiences that are more likely to contribute to adolescent development [46,48]. Interest is the opposite of boredom, implies cognitive growth, and promotes intrinsic motivation. Participation in intrinsically motivated activities has been associated with promoting self-reflection, experiencing well-being, and greater intrinsic values [25,46,49,50,51]. Although intrinsic motivation is often mentioned in the context of leisure activities and associated with developmental benefits, it is unrealistic to expect that participation in leisure activities is always intrinsically motivated. In particular, adolescents undergoing identity development rely heavily on relationships with others. In addition to intrinsic motivation, identified motivation has also been cited as a positive form of motivation and is positively related to adolescent well-being [13]. Moreover, the outcomes associated with introjected leisure motivation can be both positive and negative, as introjected motivation in adolescence is often associated with peer influence or pressure [45]. On the other hand, amotivation or external motivation is associated with negative outcomes, although there is not much literature in the leisure field to support this with empirical evidence [52]. In addition, interest and intrinsic motivation are also associated with the feeling of flow, a state of consciousness in which one loses track of time and becomes completely absorbed in the action. Flow promotes positive outcomes in youth development, including initiative, self-efficacy, and competence [46,53].

Science has long provided us with guidelines for developing evidence-based prevention interventions that can help society prevent youth involvement in risky behaviors and/or behavior problems and create opportunities for positive youth development. However, there are still some key areas that need further research as there is very limited or conflicting evidence of effectiveness, such as SLA [54]. Given that the lifestyles and leisure activities of today’s youth have changed, research is needed on how to encourage youth to proactively engage in various forms of SLA.

The purpose of this article is to examine, from the perspective of adolescents, what contributes to the adolescent’s frequency of participation in SLA. More specifically, the aim is to identify the unique contribution of adolescents’ perceptions of context, motivational factors, and the experience of participating in SLA to the frequency of adolescents’ participation in SLA.

## 2. Materials and Methods

### 2.1. Sample

The study aimed to include the entire student population (who agreed to participate in the study) of all regular high schools in Krapinsko-zagorska County, Croatia. The entire student population of all regular high schools was included to ensure variability in adolescents’ participation in SLA. Since the research was conducted with an online questionnaire during the pandemic COVID-19, it was expected that the sample would be rather scattered. The plan was to ensure a sufficient number of participants and sufficient variability to meet the stated objectives of the study based on the data collected. A total of 9 high schools were included in the sample. The sample included students from three Croatian high school education programs: gymnasium program, three-year educational schools, and four-year educational schools. The three-year and four-year programs prepare students for employment in specific professions after graduation, while the gymnasium program is intended for students pursuing higher education.

A total of 4246 students are enrolled in the Krapinsko-zagorska County area for the 2020/2021 school year. A total of 2977 students entered to the survey, representing 70.11% of the total student population in Krapinsko-zagorska County, Croatia. Of the total number of students who entered to the online survey, 82 students refused to participate in the survey, while 72 students withdrew their participation at the beginning of the survey. Ultimately, the sample consists of 2823 (66.48%) students from Krapinsko-zagorska County, Croatia.

The aim of the study was to include 1st, 2nd, 3rd and 4th grade students from all regular high schools in Krapinsko-zagorska County who participate in SLA. For the purposes of this study, only students who indicated that they participated in SLA were included in the sample (*N* = 1607, 57%). Only students who reported having participated in SLA answered the instruments regarding frequency of participation in SLA and context, motivational factors, and experiences with participation in SLA.

Students were asked to select one SLA that they attend (or attended prior to the restrictions due to the COVID 19 pandemic) that is most important to them and that they participate in for at least 1 h per week. The activity categories regarding students’ participation in SLA are shown in Table 1.

Other activities included those where students did not name an activity or indicated an activity that was not a structured leisure activity (e.g., going for a walk, hanging out with friends…). A total of 1431 students who completed a questionnaire were recruited for this study. Participants were between 14 and 21 years old (M = 18.87, SD = 1.23). A total of 44.8% of the participants were female, while 2.4% of the participants did not want to provide information about their gender (In this study, we use the term "gender" rather than “sex” to recognize that the relation of male and female identities and associations with adolescents' participation in SLA likely reflects a complex interplay of genetic, environmental, and sociocultural influences.). In a professional 2- or 3-year educational program 20.7% students were enrolled, 52.7% were enrolled in a professional 4- or 5-year educational program, and 26.6% were enrolled in a gymnasium program. Participants were also approximately evenly distributed among first, second, and third years of programs, with a lower number of fourth- and fifth-year students due to the presence of 2- and 3-year school programs in the sample. The socio-demographic characteristics of the participants who took part in SLA are shown in Table 2.

### 2.2. Research Process

Ethical approval for the study was obtained from the Ministry of Science and Education and Ethical Committee of the Faculty of Education and Rehabilitation Sciences University of Zagreb. The research team organized meetings with school principals and school counsellors to obtain their consent and partnership. Letters were sent to inform parents that the study was taking place. According to the Ethical Codex for Research with Children [55], adolescents who are 14 years old can give their consent independently.

The research was conducted in the period from March to May 2021. The research was conducted by filling out an online questionnaire in the computer classrooms or on the tablets of the schools or through the personal mobile phones of the research participants in cooperation with the professionals in the high schools of Krapinsko–Zagorska County, Croatia. In the schools where online classes take place, the research team, with the help of the professionals, organized the completion of the questionnaires on the platform through which students usually participate in online classes.

The survey was conducted using the online tool Survey Monkey. Time allotted for filling out the questionnaire was 45 min. Before completing the questionnaire, study participants indicated online whether they agreed to participate in the study. Consent to participate in the study included information about the research and its aims, the methods of data processing, the rights and protection of participants and possible risks, as well as the procedures for protecting personal data (ensuring anonymity in relation to the IP address from which the link was accessed) and the possibilities of being informed about the results of the study. Before administration of the questionnaire, participants were informed that their participation is anonymous and voluntary and that they are free to quit any time they wish.

### 2.3. Measures

The measurement instruments in English underwent a double translation, i.e., the official translator translated the instruments into Croatian and the translation was then translated into English by the research team. The two English versions of the text were then compared. If any of the variables lost their meaning, the translation into Croatian was revised. To verify the suitability and comprehensibility of the measurement instruments and to determine the exact time of filling them out, the instruments were filled out by the graduate students of social pedagogy at the Faculty of Education and Rehabilitation Sciences at the University of Zagreb before the research. After the students completed the instruments, they were asked for feedback on the instruments, i.e., on their understanding of the individual particles in the instruments. In relation to their feedback, individual variables were ambiguously reformulated.

#### 2.3.1. Questionnaire of Youth Leisure Time

To meet the needs of the project, questionnaire (15 items) were created on the following topics: the amount of leisure time youth have during the week and on weekends; the activities youth typically spend their leisure time doing; the frequency of participation in SLA (a) from January 2019 to January 2020—before the pandemic COVID 19 and (b) due to the pandemic restrictions COVID 19—2020 and early 2021); the types of SLA in which youth participate; changes in patterns of participation in SLA due to the pandemic COVID 19; payment for participation in SLA in which youth participate; inability to participate in SLA due to the pandemic COVID 19. In the analysis presented in this article, we included the question about the frequency of participation in SLA prior to the pandemic COVID 19. Students estimated frequency of participation during the week (Monday to Friday) and on weekends (Saturday and Sunday). Finally, a continuous variable—frequency of adolescents’ participation—was created (α = 0.68) to indicate the frequency of students’ participation in SLA on a monthly basis (from 0 to 28 times).

#### 2.3.2. The Context of Participation in Leisure Activities 

*The context of participation in leisure activities* (developed by Belošević & Ferić [56] was created for the needs of the project based on the theoretical research model (LACE model [2,45]. The questionnaire consists of 11 items and includes questions about emotional and physical safety during participation in leisure activities, opportunities for creativity, sense of belonging, and relationships with leaders and other participants during participation in leisure activities. Students were asked to read the items and to indicate their level of agreement on a 5-point scale (1 = strongly disagree; 5 = strongly agree). The conducted factor analyses (Exploratory Factor Analysis & Confirmatory Factor Analysis) revealed that the questionnaire consists of 3 factors: safety, opportunities, and relationships [56]. The scale score consists of 3 means (for each subscale). Below are examples of the items for each subscale:

- safety (three items, α = 0.84; e.g., “I feel emotionally safe when I do this activity”);
- opportunities (five items, α = 0.92; e.g., “The leader of this activity clearly tells me what I need to improve”);
- relationships (three items, α = 0.90; e.g., “I am satisfied with relationship with the leader of activity”).

#### 2.3.3. The Free Time Motivation Scale for Adolescents (FTMSA) 

*The Free Time Motivation Scale for Adolescents (FTMSA)* [57] was developed to assess motivation to participate in leisure activities and is suitable for use in a sample of adolescents. The scale is used to assess five types of motivation—amotivation, external, introjected, identified, and intrinsic. For each item, participants were asked to indicate their level of agreement on a 5-point scale (1 = strongly disagree; 5 = strongly agree). The scale score consists of 5 means (for each subscale) indicating the degree of motivation to participate in leisure activities. Below are examples of the items for each subscale:

- amotivation (four items, α = 0.89; e.g., “I don’t know why I do my free time activities, and I don’t really care.”);
- external motivation (five items, α = 0.77; e.g., “I do what I do in my free time because my parents expect me to”);
- introjected motivation (five items, α = 0.77; e.g., “I do what I do in my free time because I want to impress my friends”);
- identified motivation (four items, α = 0.83; e.g., “I do what I do in my free time because it is important to me”);
- intrinsic motivation (four items, α = 0.94; e.g., “I do what I do in my free time because I want to have fun”).

#### 2.3.4. The Youth Experience Survey (YES) 

*The Youth Experience Survey (YES*) [58] was developed to survey high school-school aged youth about their developmental experiences in an extracurricular activity or community-based program. The instrument was designed for use with multiethnic youth and for use across a wide range of youth programs and activities. The YES 2.0 contains 70 items covering six domains of personal and interpersonal developmental experiences and five domains of negative experiences. For each item, respondents’ rate whether they had a particular experience during their recent involvement on a 4-point scale ranging from not at all (1) to yes, definitely (4). Scale scores are computed as averages; thus, scale values range from 1 to 4, with 4 representing the highest possible rate of occurrence. The YES inventories experiences in domains of personal and interpersonal development. Personal development experiences are represented by three scales on the YES scales: Identity experiences, Initiative experiences and Emotion regulation experiences. Interpersonal development experiences are also represented by three scales: Teamwork and social skills experiences, Positive relationship experiences, and Adult networks and social capital experiences. The YES also includes five scales dealing with negative experiences: Stress, Inappropriate adult behavior, Negative peer influences, Social exclusion, and Negative group dynamics. Below are examples of the items for each subscale:

- identity experiences (six items, α = 0.83; e.g., “Started thinking more about my future because of this activity”);
- initiative experiences (refers to goal setting, effort, problem solving, and time management; 12 items, α = 0.93; e.g., “Learned to consider possible obstacles when making plans”);
- emotion regulation experiences (four items, α = 0.87; e.g., “Learned about controlling my temper”);
- teamwork and social skills experiences (ten items, α = 0.93; e.g., “Became better at sharing responsibility”);
- positive relationship experiences (ten items, α = 0.85; e.g., “Learned I had a lot in common with people from different backgrounds”);
- adult networks and social capital experiences (seven items, α = 0.85; e.g., “This activity opened up job or career opportunities for me”);
- stress (three items, α = 0.84; e.g., “This activity interfered with doing things with family”);
- inappropriate adult behavior (four items, α = 0.93; e.g., “Adult leaders in this activity are controlling and manipulative”);
- negative peer influences (four items, α = 0.90; e.g., “Felt pressured by peers to do something I didn’t want to do”);
- social exclusion (three items, α = 0.85; e.g., “I felt left out”);
- negative group dynamics (three items, α = 0.83; e.g., “Was discriminated against because of my gender, race, ethnicity, disability, or sexual orientation”).

### 2.4. Statistical Analysis

The data were analyzed using IBM SPSS 26.0 (IBM, Armonk, NY, USA) predictive analytical software. Descriptive statistics were used to determine sample characteristics, including means and standard deviations, and skewness and kurtosis. The statistical significance, general strength, and direction of the relationships between the examined variables were analyzed by Pearson’s correlation coefficients. For the main analyses, a hierarchical regression analyses were conducted to examine the unique contribution of adolescents’ perceptions of context, motivational factors, and the experience of participating in SLA to the frequency of adolescents’ participation in SLA.

As a preliminary step, the requirement of verifying multicollinearity i.e., determining whether the predictors are too highly correlated with each other, has been met. VIF values exceeding 10 are considered problematic as they suggest strong linear correlation of predictors [59]. Analysis showed absence of multicollinearity given the fact that the analysis has yielded VIF values lower than 3. In all cases, differences were significant when *p* < 0.05.

## 3. Results

A total of 42.61% of students reported that they never spend their leisure time doing SLA after school or on weekends. Students who responded that they never spend their leisure time doing SLA were asked the reasons why they do not participate. The most common reasons participants gave for not participating in SLA after school or on weekends were financial reasons, difficulty arranging transportation, lack of choice of activities in the area of living, lack of opportunities to participate in activities in the area of living, lack of interest to participate, lack of motivation to participate, and burden of school obligations and practice. The following are some quotes from students: “I do not feel the need to participate.”, “School and studying take up too much of my time.”, “Lack of opportunities, since I live in a smaller town, there are not many activities I can participate in that interest me.”, “I will be graduating this year and just do not have time for other activities. I spend all my time preparing for graduation. Additionally, we have a lot of exams at school, which makes everything even harder for me. “

Descriptive statistics (number of participants, means, standard deviations, skewness, kurtosis) were calculated for all variables studied when appropriate. The descriptive parameters of the variables studied are presented in Table 3.

Overall, 57% of survey respondents reported participating in structured leisure activities. The activity categories regarding student participation in SLA are shown in Table 1. Most students participated in sports activities (team—36.6%; individual—23.1%), followed by performance and fine arts (21%), community-oriented (5%), and educational activities (3.3%).

Pearson correlation coefficients were calculated between gender, perceptions of context (safety, opportunities, relationships), motivational factors (amotivation, external, introjected, identified and intrinsic motivation), experiences of participation in SLA (developmental experiences—identity experiences; initiative experiences; emotion regulation experiences; teamwork and social skills experiences; positive relationship experiences; adult networks and social capital experiences; and negative experiences-stress; inappropriate adult behavior; negative peer influences; social exclusion; negative group dynamics) and the frequency of adolescents’ participation in SLA (see Table S1 in Supplementary Materials). The level of statistical significance was set at *p* < 0.05. The results show that girls perceive the context (opportunities and relationships) more positively, have more pronounced intrinsic motivation, and are more likely to report that the experience of participating in SLA contributed to their experiences of identity, experiences of teamwork and social skills, and positive relationship experiences. On the other hand, boys have more pronounced external and introjected motivation and are more likely to estimate that participation in SLA has contributed to experiences of stress, inappropriate adult behavior, negative peer influences, social exclusion, and negative group dynamics. At the same time, boys are more likely to participate frequently in SLA. Regarding perceptions of context, results show that perceptions of context (safety, opportunities, relationships) are positively correlated with introjected, identified, and intrinsic motivation and negatively correlated with amotivation and external motivation to participate in SLA. Perceptions of context (safety, opportunities, relationships) are positively correlated with the developmental experiences (identity experiences; initiative experiences; emotion regulation experiences; teamwork and social skills experiences; positive relationship experiences; adult networks and social capital experiences) of participating in SLA and negatively correlated with the negative experiences (stress; inappropriate adult behavior; negative peer influences; social exclusion; negative group dynamics) of participating in SLA. At the same time, perceptions of context (safety, opportunities, relationships) are positively correlated with frequency of participation in SLA. In terms of motivation to participate in SLA, the results show that amotivation is positively related to negative experiences of participation in SLA, while it is negatively related to developmental experiences of participation in SLA. At the same time, amotivation is negatively related to the frequency of participation in SLA. Moreover, external and introjected motivation are positively related to developmental experiences, negative experiences of participation in SLA, and frequency of participation in SLA. At the same time, identified and intrinsic motivation are positively related to developmental experiences in participating in SLA and frequency of participation in SLA, and negatively related to negative experiences in participating in SLA. Developmental experiences (identity experiences; initiative experiences; emotion regulation experiences; teamwork and social skills experiences; positive relationship experiences; adult networks and social capital experiences) and stress are also positively related to frequency of participation in SLA.

The highest correlation coefficient between predictor variables was between inappropriate adult behavior and negative peer influences (r = 0.80, *p* < 0.05), inappropriate adult behavior and social exclusion (r = 0.84, *p* < 0.05), negative peer influences and social exclusion (r = 0.82, *p* < 0.05), negative peer influences and negative group dynamics (r = 0.83, *p* < 0.05), and social exclusion and negative group dynamics (r = 0.82, *p* < 0.05), indicating multicollinearity. The multicollinearity is not a problem here as these three predictors did not contribute to the variance (see Table S1 in Supplementary Materials).

To gain insight into the unique contribution of the frequency of adolescents’ participation in SLA, a hierarchical regression analysis was conducted (Table 4). The predictors were introduced in four steps: The first block of predictors consisted of a socio-demographic variable (gender) as a control variable, the second block consisted of variables of adolescents’ perceptions of the context (safety, opportunities, relationships), the third block added factors of motivation (amotivation, external, introjected, identified, and intrinsic motivation), and the fourth block consisted of adolescents’ experiences of participation in SLA (developmental experiences—identity experiences; initiative experiences; emotion regulation experiences; teamwork and social skills experiences; positive relationship experiences; adult networks and social capital experiences, and negative experiences—stress; inappropriate adult behavior; negative peer influences; social exclusion; negative group dynamics). The set of predictors proved to be statistically significant in explaining adolescents’ frequency of participation in SLA (F (20,995) = 17.321, *p* < 0.01).

Each of the blocks individually made a statistically significant contribution to explaining adolescents’ frequency of participation in SLA (*p* < 0.01). In the first step of the hierarchical regression analysis, the variable gender was statistically significantly negatively associated with adolescents’ participation in SLA (β = −0.13, *p* < 0.01), i.e., it explained 5.1% of the variance of the criterion variable. In the second step, the variables of adolescents’ perceptions of the context (safety, opportunities, relationships) explained 11.4% of the variance in adolescents’ frequency of participation in SLA. The variable safety (β = 0.25, *p* < 0.01) contributed significantly and positively to explaining the variance of adolescents’ frequency of participation in SLA, while the variables opportunities and relationship were not statistically significantly associated with the criterion variable. Thereafter, the third block explained an additional 8.3%. Amotivation (β = −0.20, *p* < 0.01) was negatively associated with the criterion variable, while external (β = 0.10, *p* < 0.01), introjected (β = 0.10, *p* < 0.01), and intrinsic motivation (β = 0.14, *p* < 0.01) were significantly and positively associated with adolescents’ frequency of participation in SLA. The last block presented the factors of adolescents’ experience of participation in SLA. The last block explained additional 4.3%.

Identity experiences (β = 0.12, *p* < 0.05), initiative experiences (β = 0.15, *p* < 0.01), and stress experiences (β = 0.12, *p* < 0.05) contributed significantly and positively to explaining variance in adolescents’ frequency of participation in SLA, whereas experiences with negative peer influences (β = −0.13, *p* < 0.05) were statistically negatively associated with the criterion variable. Experiences with emotion regulation, experiences with teamwork and social skills, positive relationship experiences, experiences with adult networks and social capital experiences, inappropriate adult behavior, social exclusion, and negative group dynamics were not statistically significantly associated with the criterion variable. However, the introduction of predictors in the final step of the analysis resulted in a decrease in magnitude and a loss of statistical significance of the regression coefficient for the estimate of the influence of introjected motivation (β = 0.07, *p* > 0.05), possibly due to intercorrelations of this variable with the other factors of motivation (see Table S1 in supplementary Materials).

The total amount of variance explained in adolescents’ frequency of participation in SLA is 29.1%, and significant predictors are gender (β = −0.23, *p* < 0.01), safety (β = 0.10, *p* < 0.05), amotivation (β = −0.16, *p* < 0.01), external motivation (β = 0.08, *p* < 0.05), intrinsic motivation (β = 0.12, *p* < 0.01), identity experiences (β = 0.12, *p* < 0.05), initiative experiences (β = 0.15, *p* < 0.01), stress (β = 0.12, *p* < 0.05), and negative peer influences (β = −0.13, *p* < 0.05). Gender, amotivation, intrinsic motivation, and initiative experiences are significant at 1% risk (*p* < 0.01), while all other predictors are significant at 5% risk (*p* < 0.05). Furthermore, we can conclude that the strongest predictors of adolescents’ frequency of participation in SLA are amotivation, initiative experiences, and negative peer influences, in addition to gender as a control variable. All predictors are positively correlated with the criterion variable except for gender, amotivation, and negative peer influences.

## 4. Discussion

The way young people spend their leisure time today has changed. More and more of them spend their leisure time with gadgets, and fewer and fewer young people engage in sports or cultural activities [6,7]. The results show that 42% of students reported that they never participate in SLA after school or on weekends. In addition to the expected reasons such as financial reasons, difficulties in organizing transport and lack of opportunities to participate in activities in their place of residence (characteristics of rural areas), students also cited lack of interest in participating, lack of motivation to participate and the burden of school commitments as reasons.

Based on the findings of previous research, it is important to understand the context, motivation and experiences of participation in leisure activities if we are to understand the effects of structured leisure on positive adolescent development [25,44]. In the context of this study, a first step toward this understanding was to determine the unique contribution of adolescents’ perceptions of context, motivational factors, and experiences of participating in SLA to their frequency of participating in SLA. The following analysis addresses the contribution of frequency of participation, perceptions of context, motivational factors, and experiences of participation in SLA in explaining adolescents’ risk behaviors.

The results showed that gender, perception of context, motivational factors, and experience of participating in SLA explained 26.9% of the frequency of adolescents’ participation in SLA. Specifically, the results suggest that boys and adolescents who perceive the context of participation as safe, are externally or intrinsically motivated to participate, and perceive that participation in SLA has contributed to their identity development and experiences of initiative, as well as their experiences of stress, are more likely to participate frequently in SLA. On the other hand, these findings indicate that girls and adolescents who are unmotivated to participate and who experience negative peer influences during their participation in SLA are less likely to participate frequently in SLA. The findings of this study in relation to gender differences in frequency of participation are consistent with the earlier study by Sylvia-Bobiak & Caldwell [60], although some studies have shown that the extent of participation in relation to gender depends on the type of leisure activity [61,62].

Participation in organized leisure activities can be developmentally beneficial during adolescence, for example, by reducing stress levels [27,63,64]. However, adolescents are often over-scheduled and sometimes participating in organized leisure activities can be stressful to balance with other aspects of life. Some adolescents participate in multiple activities, and balancing multiple leisure activities can also be challenging and stressful [27,65], which is partially consistent with the findings of this study showing that stressful experiences during participation in SLA contribute to the frequency of participation in SLA.

In relation to other findings of this study, we can conclude that creating an emotionally and physically safe environment and designing and implementing activities that promote identity development and initiative experiences (this refers to goal setting, effort, problem solving, and time management in this study) are important steps to encourage more youth to participate in SLA. We can assume that this will also increase intrinsic, identified, and even external motivation to participate in SLA. In this way, we not only promote youth participation in SLA, but also the characteristics of the context and experience of participating in SLA, which have been associated with positive youth development in numerous studies [10,13,25,41,42,44,47]. In some ways, the findings presented in this paper provide guidance on what can be done to improve youth participation in SLA.

Research has shown that participation in SLA can lead to positive developmental outcomes [17,18,22,24,26,27,28,29,30,31,32,33,34,35,36,37,38,64]. To encourage young people to participate in SLA, it is important to think much more broadly than just the setting of the activities themselves. Some of the features of SLA that promote positive youth development are presented in this paper, particularly in relation to the characteristics of the context, young people’s experiences of participation and motivation. However, there are other studies that find, for example, an association between social support and increased and sustained participation in sport and active leisure [66,67,68,69,70]. Certainly, further research is needed to understand the direction and extent to which elements may be an outcome or motivator for leisure participation in general. This statement is supported by the United Nations Office on Drugs and Crime and the World Health Organization [54], who note that in the areas of extracurricular activities, sports, and other SLA, there is very little evidence of effectiveness, or the evidence is weak and even contradictory. There is a need for an overall framework to better understand the pathways through which SLA contribute to the positive youth development and the prevention of youth risk behaviors.

### Limitations

This study has certain limitations. First, the study was conducted during the pandemic COVID 19 due to which certain restrictions on gatherings and events were in place. These measures had a significant impact on the context of leisure time. The study was conducted in the spring of 2021, when many leisure activities had already resumed and/or alternative ways had been found to maintain them. At the same time, the research team took this into account by asking questions in the survey about the time before the pandemic COVID 19 and the time of restrictions imposed by the pandemic COVID 19. At the same time, answering certain questions retrospectively (the period before the COVID 19 pandemic) could affect the accuracy and precision of study participants’ responses.

Furthermore, it was planned to cover the entire student population (who had agreed to participate in the study) of all regular high schools in Krapinsko-zagorska County, Croatia. In the end, 66.48% of the entire student population was included in the survey, while 33.52% of the students did not participate in the survey at all for the following reasons: They refused to participate in the survey, or withdrew their participation at the beginning of the survey, or they were not present in class (live or online) during the survey (the survey was conducted during the COVID 19 pandemic, when students were frequently absent from class due to “self-isolation” measures and/or due to other instructional modalities). Although the entire student population was not included, the goal of the study was met because the entire student population was included to ensure variability in adolescents’ participation in SLA. At the same time, the research team had anticipated a wider dispersion of the sample. However, the results cannot be generalized to the entire high school population. Future research should include a representative sample that allows for generalization of conclusions to the high school population. This primarily relates to data associated with epidemiological studies. However, in the context of testing analytic models, sample representativeness is not required.

In addition, adolescents’ self-assessment was used as the only source of information for measurement and a group survey was conducted, so social pressure and the possibility of giving socially desirable answers must be taken into account. In this study, this risk was addressed by conducting the study under the guidance of professional staff who gave clear instructions to the students at the beginning of the study and ensured the confidentiality of the data. At the same time, anonymity and confidentiality in the study were achieved by completing the questionnaire online. This allows for greater anonymity as the data are entered directly into the database and cannot be linked to the participants.

Although conducting the survey online has certain limitations, an attempt was made to circumvent most of them in the following ways: A group survey was organized; students could access the questionnaire only once; no personal information was requested from students; students could return to the previous question at any time during the completion of the questionnaire and change the previously given answer; at the end of the survey, students were left the contact address of the research team so that they could contact them if they had further questions about the survey or were upset by the completion of the questionnaire.

## 5. Conclusions

Leisure is an important part of everyone’s life, especially a young person. It can be considered to be an important context that enables the development of competences, such as identity development and initiative, i.e., goal setting, effort, problem solving, and time management. It could also enable the development of prosocial values in young people and can promote positive youth development. However, leisure can also be a context that fosters involvement in risk behaviors and/or the development of behavioral problems in young people. For SLA to effect change in behavioral outcomes, it is necessary to invest resources in the design and elements of interventions based on evidence of effectiveness, i.e., using empirical evidence and theories of change at the intervention development stage to achieve the intended outcomes. In summary, it is important to focus on creating opportunities and educating all key stakeholders in the community about the importance of investing in quality leisure. This will allow youth to experience quality leisure that will positively impact their development and prevent the occurrence of risk behaviors and problems among youth.

## Figures and Tables

**Table 1:** Number of students reporting their current participation in SLA (*N* = 1607).

Krapinsko-Zagorska County	
*N* (%)	1607
**Activity Category, *N* (%)**	
Sports—team	588 (36.6)
Sports—individual	372 (23.1)
Performance and fine arts (Musical, Performance, Art clubs)	337 (21)
Educational	53 (3.3)
Community-oriented	81 (5.0)
Other (unstructured activities)	176 (11)

**Table 2:** Socio-demographic characteristics of participants (sample—students participating in SLA) (*N* = 1431).

Krapinsko–Zagorska County							
*N* (%)	1431	*N* (%)	1431	*N* (%)	1431	*N* (%)	1431
Gender, *N* (%)		Age, *N* (%)		Grade, *N* (%)		High School Program, *N* (%)	
Female	641 (44.8)	14	1 (0.1)	First grade	426 (29.8)	Two or three-year vocational program	296 (20.7)
Male	756 (52.8)	15	88 (6.1)	Second grade	420 (29.4)	Four or five-year vocational program	754 (52.7)
Didn’t declare about gender	34 (2.4)	16	425 (29.7)	Third grade	327 (22.9)	Gymnasium program	381 (26.6)
		17	399 (27.9)	Fourth grade	235 (16.4)		
		18	296 (20.7)	Fifth grade	23 (1.6)		
		19	191 (13.3)				
		20	22 (1.5)				
		21	9 (0.6)				

**Table 3:** Descriptive statistics of study variable.

Variable	*n*	M	SD	Skewness (S.E.)	Kurtosis (S.E.)	α
Safety	1230	3.62	1.06	−0.57 (0.07)	−0.23 (0.13)	0.84
Opportunities	1216	3.87	1.05	−0.92 (0.07)	0.22 (0.14)	0.92
Relationships	1212	3.94	1.10	−0.90 (0.70)	0.00 (0.14)	0.90
Amotivation	1345	1.46	0.78	1.85 (0.06)	3.00 (0.13)	0.89
External motivation	1336	1.76	0.82	1.09 (0.06)	0.52 (0.13)	0.77
Introjected motivation	1319	2.48	0.93	0.33 (0.06)	−0.44 (0.13)	0.77
Identified motivation	1333	3.45	1.01	−0.73 (0.06)	−0.07 (0.13)	0.79
Intrinsic motivation	1316	4.24	1.06	−1.62 (0.06)	1.85 (0.13)	0.94
Identity experiences	1310	2.64	0.76	−0.06 (0.06)	−0.71 (0.13)	0.83
Initiative experiences	1288	2.82	0.74	−0.28 (0.06)	−0.51 (0.13)	0.93
Emotion regulation experiences	1302	2.72	0.86	−0.14 (0.06)	−0.89 (0.13)	0.87
Teamwork and social skills experiences	1228	2.83	0.77	−0.25 (0.07)	−0.59 (0.14)	0.93
Positive relationship experiences	1230	2.62	0.73	−0.00 (0.07)	−0.61 (0.13)	0.85
Adult networks and social capital experiences	1228	2.46	0.74	0.24 (0.07)	−0.39 (0.14)	0.85
Stress	1244	1.79	0.84	1.02 (0.06)	0.27 (0.13)	0.84
Inappropriate adult behavior	1230	1.48	0.78	1.67 (0.07)	1.95 (0.13)	0.93
Negative peer influences	1237	1.60	0.80	1.38 (0.07)	1.14 (0.13)	0.90
Social exclusion	1237	1.67	0.82	1.23 (0.70)	0.71 (0.13)	0.85
Negative group dynamics	1234	1.60	0.79	1.36 (0.07)	1.14 (0.13)	0.83
Frequency of adolescents’ participation	1365	11.94	7.29	0.28 (0.06)	−0.68 (0.13)	0.68

Note: M—mean; SD—standard deviation; K-S z—Kolmogorov–Smirnov test; α—Cronbach’s alpha.

**Table 4:** Hierarchical regression analyses with gender, adolescents’ perceptions of context, factors of motivation, and experience of participation in SLA as predictors and the frequency of adolescents’ participation in SLA as criterion.

Predictors			Frequency of Adolescents’ Participation in SLA				
			1. Step	2. Step	3. Step	4. Step	
			β	β	β	β	r_sp_
CONTEXT		Gender	−0.23 **	−0.25 **	−0.22 **	−0.23 **	−0.21 **
		Safety		0.25 **	0.17 **	0.10 *	0.06 *
		Opportunities		0.06	0.02	−0.03	−0.14
		Relationships		0.01	−0.04	−0.01	−0.00
MOTIVATION		Amotivation			−0.20 **	−0.16 **	−0.13 **
		External			0.10 **	0.08 *	0.06 *
		Identified			−0.01	−0.04	−0.03
		Introjected			0.10 **	0.07	0.05
		Intrinsic			0.14 **	0.12 **	0.07 **
EXPERIENCE	Developmental experiences	Identity experiences				0.12 *	0.07 *
		Initiative experiences				0.15 **	0.07 **
		Emotion regulation experiences				0.05	0.03
		Teamwork and social skills experiences				0.03	0.02
		Positive relationship experiences				−0.07	−0.04
		Adult networks and social capital experiences				−0.03	−0.02
	Negative experiences	Stress				0.12 *	0.07 *
		Inappropriate adult behavior				−0.07	−0.03
		Negative peer influences				−0.13 *	−0.06 *
		Social exclusion				0.08	0.04
		Negative group dynamics				0.03	0.01
			R = 0.05	R = 0.15	R = 0.239	R = 0.28	
			Rc^2^ = 0.050	Rc^2^ = 0.153	Rc^2^ = 0.233	Rc^2^ = 0.269	
			ΔR^2^ = 0.051	ΔR^2^ = 0.114	ΔR^2^ = 0.083	ΔR^2^ = 0.043	
			F (1.1014) = 54.545	F (4.1011) = 42.117	F (9.1006) = 30.908	F (20.995) = 17.321	
			*p* < 0.01	*p* < 0.01	*p* < 0.01	*p* < 0.01	

Note: structured leisure activities (SLA); * *p* < 0.05 ** *p* < 0.01; β—standardized regression coefficient; rsp—semi partial correlation coefficient; R—coefficient of multiple correlation; R^2^—coefficient of multiple determination; Rc^2^—adjusted multiple determination coefficient; ΔR^2^—proportion of variance explained by each group of predictors.

## Data Availability

The raw data supporting the conclusions of this article will be made available by the authors, without undue reservation.

## Supplementary Materials

The following are available online at https://www.mdpi.com/article/10.3390/ijerph19020877/s1, Table S1. Correlation coefficients between study variables.
