# Supplementary material for: Contribution of Leisure Context, Motivation and Experience to the Frequency of Participation in Structured Leisure Activities among Adolescents
Source: Int J Environ Res Public Health. 2022 Jan 13;19(2):877. doi: 10.3390/ijerph19020877 (PMC8775510; doi:10.3390/ijerph19020877)
Supplement: Supplementary file 1 [file ijerph-19-00877-s001.zip › ijerph-1462387-supplementary.pdf]

International Journal of
Environmental Research and Public Health

MDPI

Table S1. Correlation coefficients between study variables.

	1	2	3	4	5	6	7	8	9	10	11	12	13	14	15	16	17	18	19	20	21
1. Gender	-	0.05	0.11*	0.09*	-0.02	-0.15*	-0.15*	0.05	0.06*	0.06*	0.01	-0.00	0.06*	0.06*	-0.02	-0.13*	-0.17*	-0.17*	-0.11*	-0.17*	-0.22*
2. Safety		-	0.72*	0.64*	-0.27*	-0.01	0.23*	0.46*	0.42*	0.53*	0.55*	0.51*	0.52*	0.46*	0.42*	-0.01	-0.09*	-0.05	-0.06*	-0.03	0.27*
3. Opportunities			-	0.87*	-0.29*	-0.03	0.18	0.44*	0.45*	0.46*	0.50*	0.43*	0.53*	0.42*	0.36*	-0.09*	-0.19*	-0.16*	-0.13*	-0.12*	0.20*
4. Relationships				-	-0.31*	-0.08*	0.12*	0.43*	0.46*	0.39*	0.45*	0.38*	0.46*	0.36*	0.27*	-0.15*	-0.25*	-0.22*	-0.21*	-0.20*	0.18*
5. Amotivation					-	0.39*	0.05*	-0.23*	-0.39*	-0.22*	-0.27*	-0.22*	-0.19*	-0.14*	-0.07*	0.20*	0.27*	0.24*	0.25*	0.25*	-0.24*
6. External motivation						-	0.39*	0.16*	-0.07*	0.10*	0.02*	0.02*	0.01*	0.06*	0.11*	0.22*	0.22*	0.23**	0.20*	0.24*	0.09*
7. Introjected motivation							-	0.48*	0.30*	0.34*	0.33*	0.29*	0.21*	0.24*	0.24*	0.16*	0.06*	0.14*	0.10*	0.14*	0.22*
8. Identified motivation								-	0.65*	0.50*	0.50*	0.42*	0.32*	0.32*	0.25*	-0.10*	-0.19*	-0.16*	-0.18*	-0.15*	0.24*
9. Intrinsic motivation									-	0.36*	0.44*	0.38*	0.33*	0.27*	0.14*	-0.22*	-0.31*	-0.27*	-0.25*	-0.25*	0.25*
10. Identity experiences										-	0.75*	0.68*	0.54*	0.61*	0.54*	0.13*	0.00	0.07*	0.05	0.07*	0.31*
11. Initiative experiences											-	0.77*	0.63*	0.68*	0.55*	0.09*	-0.03	0.03	0.02	0.04	0.34*
12. Emotion regulation experiences												-	0.60*	0.65*	0.52*	0.09*	-0.01	0.04	0.03	0.04	0.29*
13. Teamwork and social skills experiences													-	0.75*	0.68*	0.20*	0.07*	0.13*	0.12*	0.14*	0.23*
14. Positive relationship experiences														-	0.68*	0.26*	0.15*	0.23*	0.20*	0.22*	0.22*

15. Adult networks and social capital experiences	-	0.44*	0.30*	0.39*	0.34*	0.36*	0.20*
16. Stress		-	0.68*	0.77*	0.72*	0.74*	0.07*
17. Inappropriate adult behavior			-	0.80*	0.75	0.84*	-0.01
18. Negative peer influences				-	0.82*	0.83*	0.01
19. Social exclusion					-	0.82*	0.03
20. Negative group dynamics						-	0.04
21. Frequency of adolescents' participation							-

Note: *$p < 0.05$.
